# Low-Loss Hollow Waveguide Fibers for Mid-Infrared Quantum Cascade Laser Sensing Applications

**DOI:** 10.3390/s130101329

**Published:** 2013-01-21

**Authors:** Pietro Patimisco, Vincenzo Spagnolo, Miriam S. Vitiello, Gaetano Scamarcio, Carlos M. Bledt, James A. Harrington

**Affiliations:** 1 CNR-IFN and Dipartimento Interateneo di Fisica, Università and Politecnico di Bari, Via Amendola 173, I-70126 Bari, Italy; E-Mails: vincenzo.spagnolo@uniba.it (V.S.); gaetano.scamarcio@uniba.it (G.S.); 2 CNR-Dipartimento di Scienze Fisiche e Tecnologie della Materia, Largo E. Fermi 5, 50125 Firenze, Italy; E-Mail: miriam.vitiello@sns.it; 3 NEST, Istituto Nanoscienze-CNR and Scuola Normale Superiore, Piazza San Silvestro 12, I-56127 Pisa, Italy; 4 Department of Material Science & Engineering, Rutgers University, Piscataway, NJ 08855, USA; E-Mails: carlos_bledt@brown.edu (C.M.B.); jaharrin@rutgers.edu (J.A.H.)

**Keywords:** hollow glass waveguide, quantum cascade laser, gas sensing, quartz enhanced photoacoustic spectroscopy

## Abstract

We report on single mode optical transmission of hollow core glass waveguides (HWG) coupled with an external cavity mid-IR quantum cascade lasers (QCLs). The QCL mode results perfectly matched to the hybrid HE_11_ waveguide mode and the higher losses TE-like modes have efficiently suppressed by the deposited inner dielectric coating. Optical losses down to 0.44 dB/m and output beam divergence of ∼5 mrad were measured. Using a HGW fiber with internal core size of 300 μm we obtained single mode laser transmission at 10.54 μm and successful employed it in a quartz enhanced photoacoustic gas sensor setup.

## 1. Introduction

Fiber-optic components show enhanced versatility with respect to bulk optics, mostly in terms of flexible beam guidance and compactness. Furthermore, fibers could also be deployed advantageously as modal wavefront filters, optical path delay length control or for multi-axial beam combining [1]. There are various fields of application for infrared fibers, each one requiring special fiber properties such as: nulling interferometry [2], high precision imaging and spectroscopy [3]. Moreover, fiber optics is a key enabling technology needed to improve the robustness and effectiveness of specific detection and calibration systems.

Optical fibers can be characterized by structure, guiding-type, and material. A simple classification by structure is: solid-core fibers, hollow waveguides and micro-structured fibers (so-called photonic crystal fibers). Waveguiding in solid-core fibers obeys the principle of total reflection of light propagating inside the core. Total reflection is caused by refractive index differences between the core and cladding materials. Fibers transmitting light of wavelength larger than 2 μm can be manufactured using glass or crystalline materials [4]. In this spectral range silica fibers transmission is limited by multi-phonon absorption [5].

## 2. Hollow Glass Waveguides

**Figure 1.**
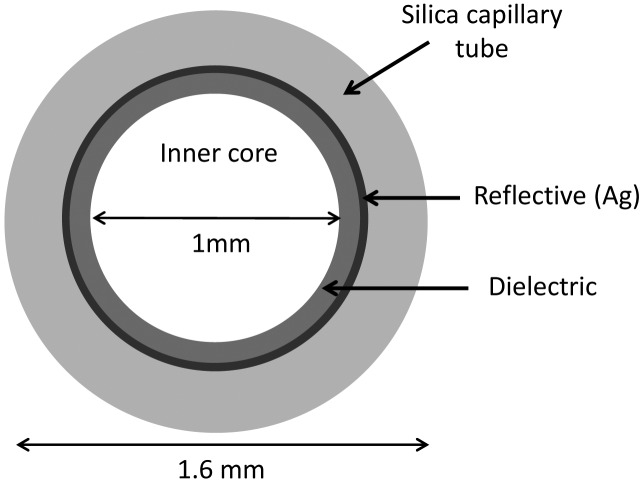
Cross-section of the cylindrical hollow waveguides. The inner part consists of a metallic Ag layer coated by a single dielectric film of AgI or polystyrene (Ps) or by AgI/Ps double dielectric film. The outer part consists of silica with UV acrylate external coating.

In hollow core glass waveguides (HGWs) light can propagate through an air core by multiple reflections on the metallic inner wall [6]. The schematic of a typical hollow guide fiber is shown in Figure 1. The main advantages are high power threshold, low insertion loss, low nonlinearity, and no end-reflection [7]. Furthermore, hollow waveguides show low beam divergence and losses are proportional to *a*^−3^, where *a* is the bore radius. Drawbacks are the high bending losses, which are proportional to R^−1^, where R is the bending radius [8]. Considering the cylindrical geometry of an hollow waveguide, the optical modes are transverse electric (TE*_lm_*) or transverse magnetic (TM*_lm_*). There are also hybrid or mixed modes designated by HE*_lm_* and EH*_lm_*. These hybrid fiber modes have a small component of the electric field along the fiber or optic axis and correspond to skew rays (a ray that does not cross the fiber axis; rather it travels in a corkscrew or helical path down the fiber). In order to reduce the overall waveguide transmission losses due to the absorption of radiation in the metallic layer, the waveguide core is additionally coated with dielectric films [9]. Actually, the losses at IR frequencies are greatly reduced by adding dielectric thin films to metallic or metallic coated waveguides. The thickness of the dielectric film chosen to minimize losses depends on the wavelength and on the refractive index of the dielectric material employed [10]. A schematic sketch of the proposed waveguide concept is reported in Figure 1.

In a hollow waveguide the total transmission losses depend on the nature and number of the propagating modes and increase dramatically for the higher order modes [6]. Therefore, it is often desirable to excite through the waveguide only the lowest-order mode and when a Gaussian beam is focused on-axis into a hollow waveguide, only the hybrid HE_1,m_ modes are excited.

Characterization of mode dispersion characteristics and propagation losses is conventionally done by employing time domain spectroscopy or bulky gas lasers. However, to address the mode stability and to evaluate the bending loss, the characterization system is required to distinguish between waveguide modes.

In the present work we report on experimental studies aiming at coupling the low loss optical modes of hollow waveguides having a metal and dielectric internal coating with high efficiency mid-IR quantum cascade lasers (QCLs), therefore prospecting a novel integrated optical system suitable for high sensitivity and high resolution spectroscopy experiments.

## 3. Experiments and Results

HWGs having lengths in the range 2–14 cm and bore diameter of 1 mm have been used to guide the optical beam of two external cavity edge-emitting mid-IR QCLs [11]. Three different dielectric coating were deposited over the internal silver layer with thickness of 1 μm: (i) AgI (0.6 μm); (ii) polystyrene (Ps, 7.7 μm); (iii) a double dielectric layer of AgI/Ps (0.6/7.7 μm). The fabrication of Ag/AgI-coated HGWs begins with a silica tubing, which has polymer (UV acrylate) coating on the outside surface. A liquid-phase chemistry technique [7] is used to deposit the Ag and the dielectric films inside the glass tubing. The first step involves depositing a silver film using standard Ag plating technology. Immediately after the silver is deposited, an iodine solution is pumped through the tubing and, through a subtraction process, a layer of AgI is formed. By controlling the concentration of the iodine solution and reaction time, an AgI film of the correct optical thickness can be deposited. To prepare silver/polystyrene (Ps)-coated hollow glass waveguides, an Ag film is first deposited within a glass substrate tube by combining a solution containing AgNO_3_ and NH_4_OH with a reducing solution containing dextrose and Na_2_EDTA [12]. The Ag film grows at a rate of approximately 1 μm/h, and Ag films were normally about 1 μm thick. The Ps films were deposited over the Ag coatings by using a liquid-flow coating process in which a Ps/toluene solution is drawn through the HGW at a constant coating velocity.

In all cases the coating is expected to reduce the mode dispersion and the attenuation of the optical modes with lower losses. Indeed, adding a thin film on the inner surface of silver wall changes the boundary conditions and can in principle reverse the dominant mode order. As a consequence, the thickness for the non-absorbing coating can enhance or suppress specific modes, if the inner coating thickness is larger than the critical value [9]:

(1) $$d=\frac{\lambda }{2\pi \sqrt{n^{2}-1}}\tan^{-1}\left[\frac{n}{\sqrt[{4}]{n^{2}-1}}\right]$$

where n is the refractive index of the dielectric film and λ is the wavelength of propagating light in the waveguide. For what concern the thickness of PS, using Equation (1), we estimate a critical value of the layer thickness of d_PS_ = 0.53 μm (λ_a_ = 5.7 μm) and d_PS_ = 1.1 μm (λ_b_ = 10.5 μm). The PC layer thickness has been set to 7.7 μm, much higher than the critical value for both wavelengths. We select this layer thickness since poor quality interference patterns in the near IR have been observed for thinner PC layers, meaning that the films are not uniform and so lower reflectance and thus larger losses are expected.

Two commercial mid-IR QCL sources based on an external cavity configuration were used for our experiments. The selected emission wavelengths are λ_a_ = 5.27 μm and λ_b_ = 10.5 μm (Daylight Solutions Inc., San Diego, CA, USA, model #21052-MHF and #21106-MHF, respectively). The average optical powers measured using a pyroelectric detector were 93 mW (λ_a_) and 65 mW (λ_b_). We tested metallic/dielectric waveguides for both wavelengths, considering that at the employed laser wavelengths, the dielectric film thickness deposited in the inner waveguide core are larger than the critical coating thickness for AgI and Ps.

The intensity profile of the propagating optical mode has been collected by means of a pyroelectric detector having a sensitive area of 1 mm × 1 mm, mounted on a compact motorized XY stage. The 2D multi-point image of the beam profile was obtained by recording the total transmitted power in the range 1–5.1 cm away from the waveguide output in the far field configuration for each scanning position, with a spatial resolution of about 0.2 μm for both XY scanning directions.

**Figure 2.**
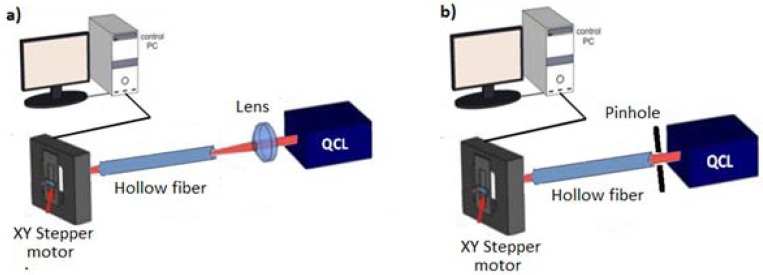
Schematics of the experimental setup. The laser beam is focused at the waveguide entrance using a ZnSe lens (**a**) or in a simple back-to-back configuration (**b**) and detected using a pyroelectric detector equipped with a XY stepper motor.

A first set of experiments were carried out coupling the QCL and the hollow waveguide by using a ZnSe lens to focus the collimated beam into a ∼100 μm spot at the waveguide entrance [13–15], as schematically shown in Figure 2(a).

The focusing lens allows to obtain an input spot size smaller than the waveguide bore diameter in order to reduce the amount of energy blocked by the waveguide walls, thus reducing the light absorption heating, which is a critical point for high-power applications.

**Figure 3.**
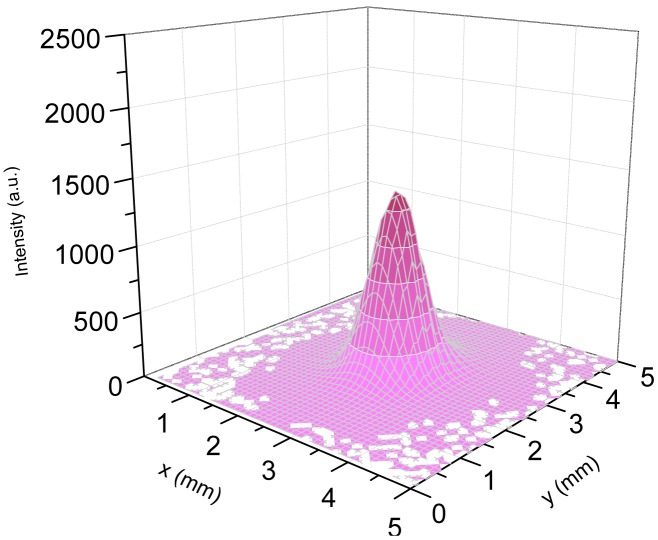
Far field spatial intensity distribution of the λ_b_-QCL. The beam profile has been measured under the experimental configuration of Figure 2(b) without any optics between the laser and the detector stage.

Figure 3 shows a representative far field 3D spatial intensity distribution measured for the λ_b_ QCL source The QCL current was set at 900 mA. The intensity distribution profile exhibits a Gaussian-like beam shape, as expected for a standard edge emitting QCL.

**Figure 4.**
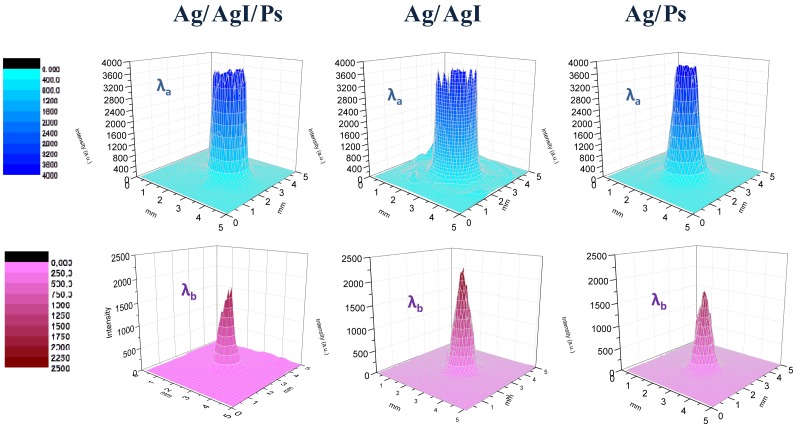
Far-Field spatial intensity distribution of the λ_a_ (**upper row**) and λ_b_ (**lower row**) upon exiting a 12 cm long and 1 mm core diameter hollow waveguide having AgI/Ps double dielectric layer and having a single dielectric film of AgI and Ps. The beam profiles have been measured by focusing the QCL beam directly at the center of waveguide by using a ZnSe lens.

Figure 4 shows the far-field spatial intensity distribution of the two employed QCLs at the exit of a 12 cm long waveguide for different internal dielectric coating configuration measured 2 cm away from the waveguide output.

**Figure 5.**
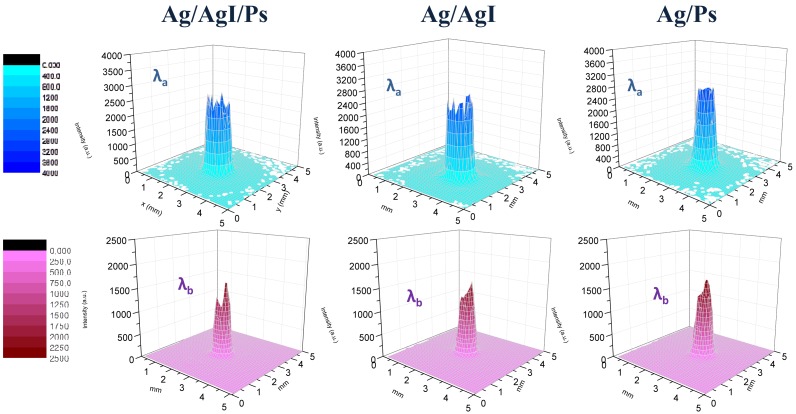
Far-Field spatial intensity distribution of the λ_a_ (**upper row**) and λ_b_ (**lower row**) upon exiting a 12 cm long and 1 mm bore diameter hollow waveguide having AgI/Ps double dielectric layer and having a single dielectric film of AgI and Ps. The beam profiles have been measured in a back-to-back configuration.

In a different experimental scheme, the QCL sources were placed directly in close contact with the fiber input with no intermediate optics, in so-called back-to-back configuration, as schematically shown in Figure 2(b). A steel circular pinhole with the same diameter of the hollow core was placed in contact with the waveguide exit to prevent detection of scattered light not propagating through the waveguide. Figure 5 shows the far-field spatial intensity distribution of the two wavelengths at the exit of a 12 cm long waveguide in back-to-back configuration measured 2 cm away from the waveguide output. The electric field intensity distribution shows that the beam shape is perfectly matched to the hybrid HE*_11_* mode since the electric field associated to the optics field at the boundary is reduced along the entire contour of the waveguide wall, meaning that the deposited inner dielectric coating thickness suppresses effectively the higher losses TE-like modes. Note that the theoretical beam profile for HE*_11_* mode in the waveguide can be approximated by the zero order Bessel function $E(r)=E_{0}J_{o}(u_{1}\frac{r}{a})$.

Despite the condition a ≫ λ is satisfied, we observed a single mode profile at the waveguide output in all investigated cases. However, it must be considered that in hollow waveguides the propagating losses for the higher order modes increase as the mode parameter squared u_lm_[6]:

(2) $$\alpha_{lm}={\left(\frac{u_{lm}}{2\pi }\right)}^{2}\frac{\lambda^{2}}{a^{3}}Re\left[\frac{n^{2}+1}{2\sqrt{n^{2}-1}}\right]$$

Thus the losses for the higher-order modes become so high that they are damped out and only the lowest-order modes can propagate.

Our results demonstrate that the HE*_11_* mode losses in an optimally designed waveguide become lower than those for TE*_01_* mode [16], even for dielectric coatings with a moderate absorption coefficient. It is worth noting that the output beam profile of hollow waveguides depends not only on the bore diameter or the propagating wavelength, but also on the quality and launch conditions (with or without the focal lens) of the input beam, as theoretically expected [6]. We found that the beam divergence is larger when the ZnSe lens is used to focalize the laser beam at the waveguide entrance, especially for the wavelength λ_a_, because additional optical modes are propagating through the straight waveguide.

## 4. Propagation Losses and Coupling Efficiency

Propagating losses in hollow waveguides are highly dependent on the launch conditions, *i.e.*, the ratio *ω*/*a*, where *ω* is the beam waist at the waveguide entrance [8]. The power coupling efficiency of the incident beam to excite each low-loss HE_1m_ waveguide mode depends critically on the beam waist to bore radius ratio. Theoretically up to ∼100% of power coupling efficiency of an incident Gaussian beam to the lowest-loss HE*_11_* mode can be obtained. At small f-numbers more power is coupled to the higher-order modes. Instead at large f-numbers the beam can be clipped by waveguide walls. Since the HE*_11_* mode has the lowest theoretical loss, it is necessary to optimize the launch conditions in order to couple most of the input beam into this mode. This is possible by changing the value of the ratio *ω*/*a* (*i.e.*, varying the focusing lens position with respect to the waveguide entrance) and maximizing the output power in order to couple most of the light in the HE*_11_* mode. Our experiments have been performed under these conditions.

The propagation losses and the coupling efficiency can be determined by making two measurements. First we measured the optical power I0 at the waveguide entrance, then the light is coupled into the fiber and the optical power IS at the output-end of the fiber is measured. Absolute values of the losses of the excited mode of the straight waveguide were determined by using the following equation:

(3) $$\text{dB}=10\log_{10}\left(\frac{{\text{I}}_{0}}{{\text{I}}_{\text{S}}}\right)$$

**Figure 6.**
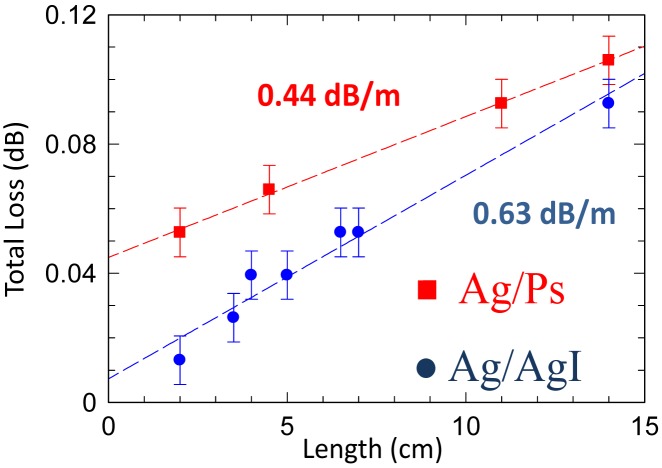
Total losses calculated from the ratio of input/output power values for the wavelength λ_a_ at the waveguide entrance/exit for Ag/AgI coatings (● symbols) and for AgPs coatings (■ symbols). The dashed lines are linear fits to the data. The reported transmission losses have been estimated from the slope of each linear fit.

The graph in Figure 6 shows the values measured by coupling hollow waveguides of different lengths (in the range 2–14 cm) with laser light at λ_a_ wavelength in back-to-back configuration.

The transmission losses increase linearly with the waveguide length. From the slope of the linear fit to the data we measured transmission losses of 0.63 dB/m for Ag/AgI coatings and 0.44 dB/m for Ag/Ps coatings. These values are nearly one order of magnitude lower that optical power attenuation measured using single-mode hollow core photonic crystal fibers made of silica glass [17].

The coupling efficiency values between the QCL source and the waveguide is extracted from the intercept at zero length of the linear fits to the data. Coupling efficiencies as high as 99.8 ± 0.2% for the AgI dielectric film and 99.0 ± 0.1% for Ps coating have been obtained. The small variation between the coupling efficiencies is due to slight differences in the launch conditions, *i.e.*, the ratio *ω*/*a*. The theoretical coupling efficiency is 99.6% [8].

## 5. Output Divergence Angle

The output divergence angle characterizes the diffraction of light leaving the waveguide. The output-beam divergence of a HGW depends critically on three factors: (i) the nature of the optical mode propagating through the guide; (ii) the wavelength and the polarization of the incoming beam; (iii) the bore diameter.

**Figure 7.**
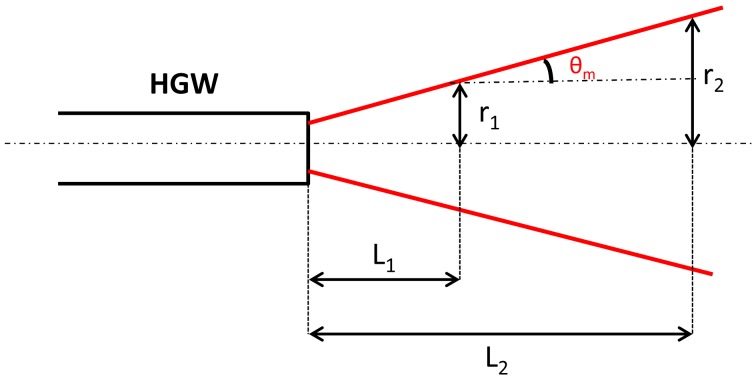
Schematics of the output divergence angle measurement.

In principle the output beam divergence can give an indication of the number of high-order mode propagating in the hollow waveguide. The HE*_1m_* modes will couple to free-space modes with a beam divergence *θ_m_* given by:

(4) $$\theta_{m}≅\sin\theta_{m}=\frac{u_{1m}\lambda }{2\pi a}$$

where *u*_1_*_m_* is a parameter which depends on the order number (*m*) of the HE*_1m_* mode. The values for *u*_1_*_m_* rapidly increase for the higher-order modes [7,9]. The schematic of the output divergence measurements is shown in Figure 7.

The output divergence angle *θ_m_* has been calculated from:

(5) $$\theta_{m}≅\tan\theta_{m}=\frac{r_{2}-r_{1}}{L_{2}-L_{1}}$$

where *r*_1_ and *r*_2_ are the radial distances at which the light intensity drops to 1/*e*^2^ of its maximum central value, measured respectively at *L*_1_ = 1 cm and *L*_2_ = 5.1 cm distance from the fiber output as measured from the far-field scans.

**Figure 8.**
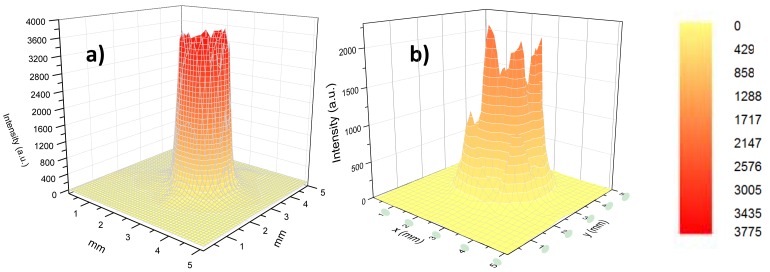
Far-field spatial intensity distribution upon exiting a 12 cm long and 1 mm bore diameter hollow waveguide having a single dielectric film of AgI and Ps at L_1_ = 1 cm (**a**) and *L*_2_ = 5.1 cm (**b**) distance from the waveguide exit.

Figure 8 shows the 3D-far field scans measured for a 12 cm long HWG with AgI dielectric internal coating and at λ_a_ propagating wavelength. From these data we extracted *r*_1_ = 0.5 mm and *r*_2_ = 0.75 mm and estimated a divergence of *θ*_m_ = 4.92 mrad. By using Equation (4), we estimated a mode parameter *u*_1_*_m_* = 2.94, in good agreement with the expected theoretical value for the first propagating mode *u*_11_ = 2.405.

Much larger values are expected for higher order mode [6], as shown in Table 1 further confirming single-mode propagation of the laser beam in the hollow waveguide and good mode coupling between the laser-input optical mode and the waveguide lowest-loss HE*_11_* mode.

## 6. Single-Mode HWG for Optical Sensing Applications

A particularly attractive application for IR-fiber optics is optical sensing. In general, silica-based optical fibers are used in a large number of sensor applications such as: chemical and radiometric sensing, fiber optic gyros, *etc.* Fewer applications for IR-fibers have been demonstrated. The main reasons are the higher losses and the difficulty to achieve single mode propagation fibers, which are typically required in spectroscopic sensors.

**Figure 9.**
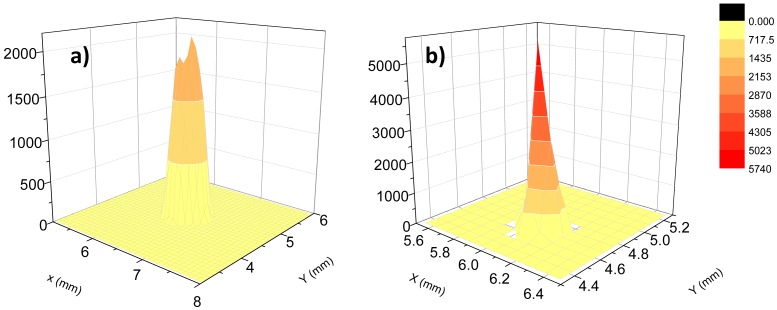
Three-dimensional single mode profile of the EC-QCL beam: exiting the hollow fiber (**a**) and at the focusing plane of the collimator (**b**). The color map, identical for both panels, is shown in right side of the figure.

For examples, in quartz enhanced photoacoustic (QEPAS) sensing, it is critical to avoid laser illumination of the acoustic detection module (ADM), since the radiation blocked by the Quartz tuning fork (QTF) and associated micro-resonator tubes creates an undesirable background when absorbed by the ADM structural elements. This background is usually several times larger than the thermal noise level of QEPAS and carries a shifting fringe-like interference pattern, which limits the detection sensitivity [18–20]. Thus, it is important to employ a mid-infrared QEPAS excitation beam of high quality and stability and if employing a fiber coupling system, single mode beam delivery is strictly required. Recently, we designed and assembled a QEPAS system fiber coupled with a single mode QCL and exploited its high performance for the detection of SF_6_ gas traces in the ppt range. Single mode laser delivery has been obtained using a hollow fiber with inner Ag–AgI coatings and an internal bore size of 300 μm, having a loss of 1 dB/m. Additional collimating optics have been designed to be attached to the output of the fiber and provide a focusing distance of 40 mm. In Figure 9 is shown the 3D laser beam profile measured using a pyroelectric camera (mod. Spiricon Pyrocam IIIC, AhrensburgGermany) at the output of the fiber (Figure 9(a)) and at the focusing plane of the collimator (Figure 9(b)). From Figure 8(b) we can estimate a focused beam waist diameter of ∼170 μm, well below the gap between the QTF prongs (∼300 μm). As a results, in the QEPAS experiments almost all (99.4%) of the laser beam coming out the collimator were transmitted through the ADM module without touching it, thus reducing the background pattern and allowing to reach detection sensitivity in the part-per-trillion (ppt) concentration range. A record normalized noise equivalent absorption coefficient for QEPAS of 2.7 × 10^−10^ cm^−1^·W/Hz^½^ was achieved [20].

## 7. Conclusions

Optical coupling between mid-IR QCLs and metallic hollow waveguides with a single or double internal dielectric coating was investigated by mid-IR far-field imaging. Single-mode propagation of the laser beam in the hollow waveguide and excellent optical mode coupling between the incoming beam and the waveguide mode have been observed. The optical losses measured for 1,000 μm bore diameter at 5.27 μm was 0.63 dB/m for Ag/AgI coatings and 0.44 dB/m for Ag/Ps coating. The output divergence angle at the Ag/AgI waveguide exit was < 5 mrad for λ = 5.27 μm, which makes it possible to use these fibers in several applications such as radiometry or chemical sensing. Single mode hollow fibers have been recently employed in a QEPAS experiments to couple the output beam of a quantum cascade laser with the acoustic detection module of the sensors. Highly focused beams were obtained, allowing an almost perfect transmission of the laser beam trough the module. This strongly reduces the sensor background signal, allowing record part-per-trillion detection limit for SF_6_.

## Figures and Tables

**Table 1.** Values for the mode parameter u_1m_ for some of the lowest-order mode.

	***l*** = **1**
*m* = 1	2.405
*m* = 2	5.52
*m* = 3	8.654
*m* = 4	11.796
